# Supplementary material for: Wastewater Metavirome Diversity: Exploring Replicate Inconsistencies and Bioinformatic Tool Disparities
Source: Int J Environ Res Public Health. 2025 Apr 30;22(5):707. doi: 10.3390/ijerph22050707 (PMC12111215; doi:10.3390/ijerph22050707)
Supplement: Supplementary file 1 [file ijerph-22-00707-s001.zip › Supplementary_Material.pdf]

Suplementary Material

Wastewater metavirome diversity: exploring replicate inconsistencies and bioinformatic tool disparities

Andre Santos1, Monica Nunes2, Andreia Filipa Silva3, Victor Pimentel1, Marta Pingarilho1, PatriciaAbrantes1, Mafalda N. S. Miranda1 , Teresa Crespo4 , Ana B. Abecasis1 , Ricardo Parreira1 and Sofia G.Seabra1,*

1 Global Health and Tropical Medicine, GHTM, Associate Laboratory in Translation and Innovation TowardsGlobal Health, LA-REAL, Instituto de Higiene e Medicina Tropical, IHMT, Universidade NOVA de Lisboa,Portugal
2 cE3c–Centre for Ecology, Evolution and Environmental Changes & CHANGE–Global Change and Sustainability Institute, Faculdade de Ciencias, Universidade de Lisboa, Campo Grande, 1749-016 Lisbon, Portugal
3 CIIMAR/CIMAR-LA, Centro Interdisciplinar de Investigacao Marinha e Ambiental, Universidade do Porto, Matosinhos, Portugal
4 iBET, Instituto de Biologia Experimental e Tecnologica, Oeiras, Portugal

* Correspondence: SGSeabra@ihmt.unl.pt

Protocol for wastewater sample processing

Viral-like particles were concentrated using organic flocculation with pre-flocculate powdered milk solution (1\% (m/v)), where 10 g of powdered milk (Conda Pronadisa, Madrid, Spain) was dissolved in 1 L of artificial sea water (Sigma Aldrich St Louis, Missouri, EUA), with the pH adjusted to 3.5 using 1M HCl. Ten mL and 100 mL of the previous solution were added to 1 L and 10 L wastewater samples, respectively (final concentration of powdered milk at 0.01\% (m/v)), and the pH was adjusted to 3.5 with 1M HCl. Subsequently, the water samples were kept under constant stirring for 8 hours at room temperature, and then for another 8 hours without stirring for the flocculation sediments to settle. The supernatants were carefully removed using a vacuum pump so as not to disturb the sediment. This sediment (about 500 ml) was then transferred to a centrifuge bottle and concentrated by centrifugation at 5500 RCF for 45 minutes at 12 °C in the J-26 Avanti® XPI Beckman Coulter centrifuge. Afterwards, the supernatant was discarded and the concentrate, which contained the viral particles, was re-

suspended in 8 mL of 0.2 M phosphate buffer with a pH of 7.5 (1:2 (v/v) mixture of 0.2 M Na_2HPO_4 and 0.2 M NaH_2PO_4). The concentrate was stored at -20 °C until further use.

SISPA Protocol

Total RNA was reverse-transcribed into cDNA using Invitrogen Superscript IV (Life Technologies, Austin, TX, USA) and a primer (A) that consisted of a 17-nucleotide specific sequence followed by a random nonamer for random priming (A-(5'-GTTTCCCAGTCACGATANNNNNNNNN-3')) [19]. The complementary strand of the cDNA was synthesized using Sequenase 2.0 (USB/Affymetrix, Cleveland, OH, USA). To generate sufficient DNA for sequencing, a pre-amplification PCR was carried out with primer B (5'-GTTTCCCAGTCACGATA-3') and AmpliTaqGold (Life Technologies, Austin, TX, USA). The PCR conditions included an initial denaturation at 95 °C for 10 minutes (hot start), followed by 30 cycles of denaturation at 94 °C for 30 seconds, annealing at 50 °C for 30 seconds, and extension at 72 °C for one minute. A final extension step at 72 °C for 10 minutes was also included. Excess primers and dNTPs were removed by cleaning and concentrating the PCR products using the Zymo DNA Clean and Concentrator kit (Zymo Research, USA). DNA concentrations were measured using Qubit 2.0 (Life Technologies, USA), and libraries for each sample were constructed using the KAPA HyperPlus Library Preparation kit (Roche, Switzerland) according to the manufacturer's guidelines.
