# Supplementary figures and images for: Wastewater Metavirome Diversity: Exploring Replicate Inconsistencies and Bioinformatic Tool Disparities
Source: Int J Environ Res Public Health. 2025 Apr 30;22(5):707. doi: 10.3390/ijerph22050707 (PMC12111215; doi:10.3390/ijerph22050707)

CZ.ID

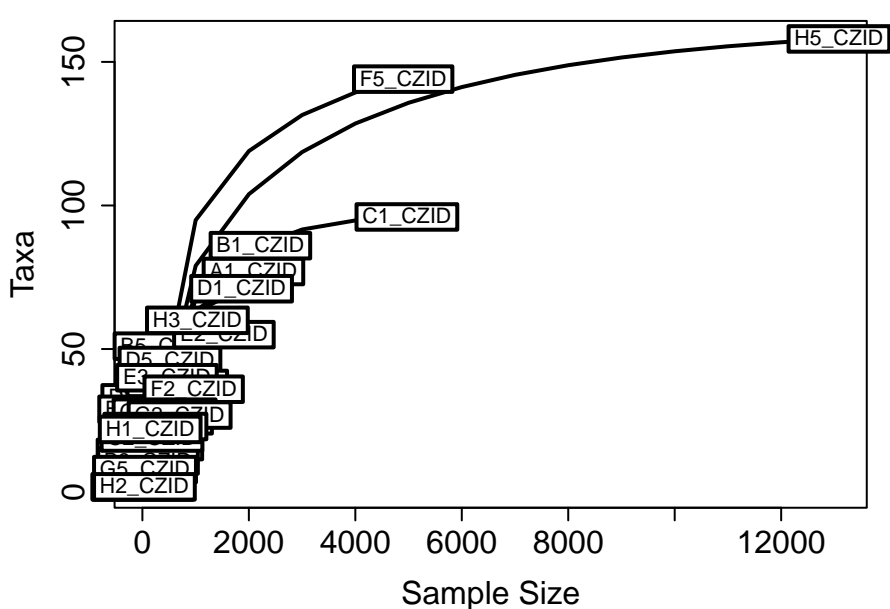

Genome Detective

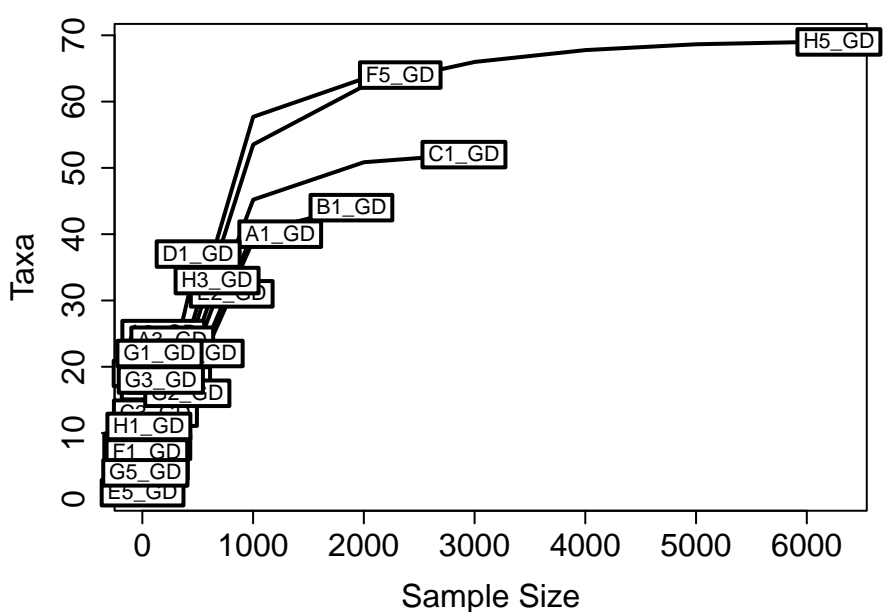

Kraken2

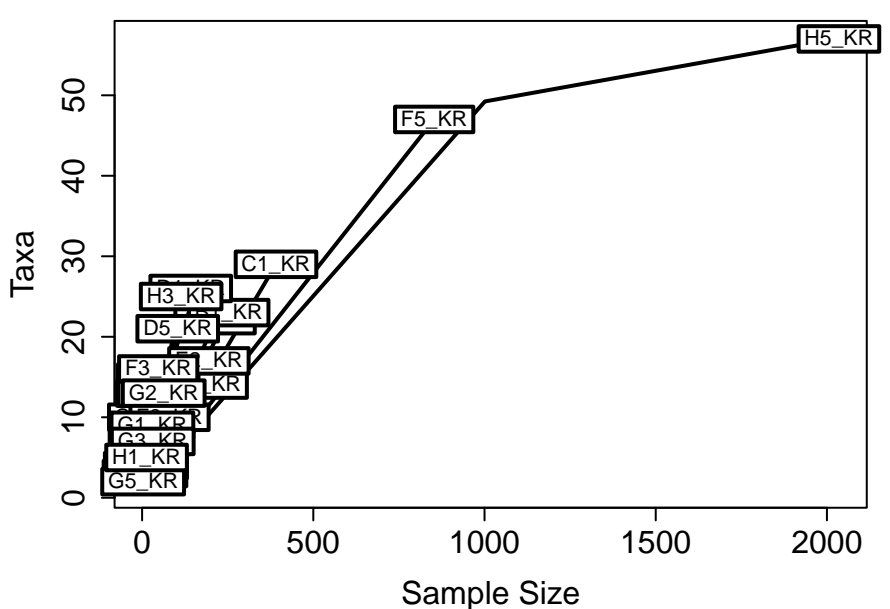

INSaFLU-TELEVIR

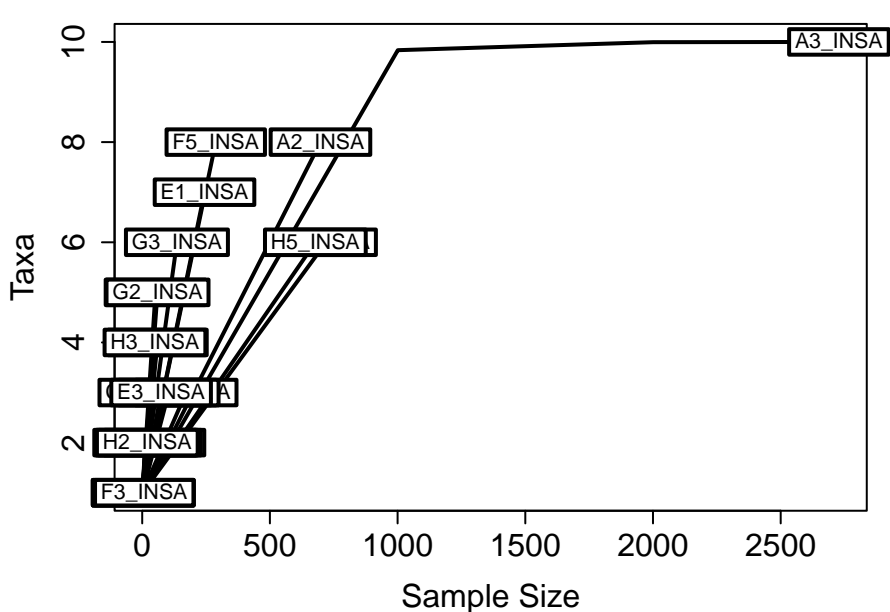

Supplement: Supplementary file 1 [file ijerph-22-00707-s001.zip › Supplementary_Figure_S1.pdf]
